# Fluorescent Magnetic Nanoparticles for Bioimaging through Biomimetic Surface Modification

**DOI:** 10.3390/ijms24010134

**Published:** 2022-12-21

**Authors:** Andrey S. Drozdov, Kristina S. Komarova, Elizaveta N. Mochalova, Elena N. Komedchikova, Victoria O. Shipunova, Maxim P. Nikitin

**Affiliations:** 1 Moscow Institute of Physics and Technology, 141701 Dolgoprudny, Moscow Region, Russia; 2 Prokhorov General Physics Institute of the Russian Academy of Sciences, 119991 Moscow, Russia; 3 Research Center for Genetics and Life Sciences, Sirius University of Science and Technology, 354340 Sochi, Russia; 4 Shemyakin-Ovchinnikov Institute of Bioorganic Chemistry, Russian Academy of Sciences, 117997 Moscow, Russia

**Keywords:** magnetite, dopamine, surface modification, hybrid materials, in vivo imaging, biodistribution

## Abstract

Nanostructured materials and systems find various applications in biomedical fields. Hybrid organo–inorganic nanomaterials are intensively studied in a wide range of areas, from visualization to drug delivery or tissue engineering. One of the recent trends in material science is biomimetic approaches toward the synthesis or modification of functional nanosystems. Here, we describe an approach toward multifunctional nanomaterials through the biomimetic polymerization of dopamine derivatives. Magnetite nanoparticles were modified with a combination of dopamine conjugates to give multifunctional magneto-fluorescent nanocomposites in one synthetic step. The obtained material showed excellent biocompatibility at concentrations up to 200 μg/mL and an in vivo biodistribution profile typical for nanosized formulations. The synthesized systems were conjugated with antibodies against HER2 to improve their selectivity toward HER2-positive cancer cells. The produced material can be used for dual magneto-optical in vivo studies or targeted drug delivery. The applied synthetic strategy can be used for the creation of various multifunctional hybrid nanomaterials in mild conditions.

## 1. Introduction

Nanomaterials and nanostructured systems emerged in the biomedical field of study in the middle of the last century and still attract a lot of attention due to their unique properties [1,2,3,4]. Several dozens of nanoformulations reached clinical application and hundreds are under preclinical studies. While the majority of clinically used systems are of organic nature, hybrid organo–inorganic systems are also under intense investigations in preclinical or early clinical stages [5,6,7]. The interest in organo–inorganic hybrid materials originates from their unique physicochemical properties and tunability of their activities. This is achieved by proper design of the systems and application of materials with desired properties [2,8,9,10,11]. Strategies for the synthesis of new hybrid materials are developing in parallel with the conceptualization and design of new systems. The collected mass of available data has shown that to achieve the successful application of synthesized materials, the procedures and reagents used during their production should be kept as safe as possible to simplify purification processes and lower their undesired bioactivities [12,13,14]. From this point of view, application of biogenic materials looks preferable due to their metabolic profiles.

Fifteen years ago, analysis of a mussel’s surface attachment mechanisms revealed that it is mediated through the adhesive foot protein, Mefp-5 (Mytilus edulis foot protein-5) rich with 3,4-dihydroxy-L-phenylalanine (DOPA) and lysine residues that bear catechol and amino groups, respectively [15]. This discovery led to the creation of polydopamine (PDA) coatings which are readily formed in mild conditions through oxidative cyclization and polymerization of dopamine by dissolved oxygen [16,17,18,19,20]. Such coatings are synthetically available, biocompatible, rich in amino groups for further modification, and have found various applications in theranostics, tissue engineering, etc. [20,21,22].

Further development of the concept shifted the attention from commercially available dopamine to dopamine derivatives and other polyphenols for surface modification. In this approach, firstly, functional monomers are synthesized by conventional organic synthesis, and then the coating is applied on the substrate’s surface. Such a two-step procedure allows separation of the `’organic” and the `’colloidal” stages to simplify the characterization and purification of the products and increase the ratio between the applied and successfully immobilized motifs. One of the benefits of such an approach is also in its versatility and opportunity to immobilize several functional groups simultaneously by deposition of a mixture of several dopamine derivatives, but this possibility has only limited representation in the scientific literature [23,24,25].

Here, we present a biomimetic approach to the creation of multifunctional hybrid systems with magneto-optical properties based on the application of dopamine derivatives. Composites were synthesized by biomimetic polydopamine formation using biocompatible magnetite nanoparticles and two organic precursors with carboxylic and fluorescent residues. Application of two different dopamine derivatives in one process allowed us to introduce a carboxyl group and fluorophore in one synthetic step in a controlled manner and to create an organo–inorganic composite material for biomedical applications. Physicochemical properties, cytotoxicity and in vivo behavior were described.

## 2. Results

### 2.1. Synthesis and Modification of Composites

**Figure 1:**
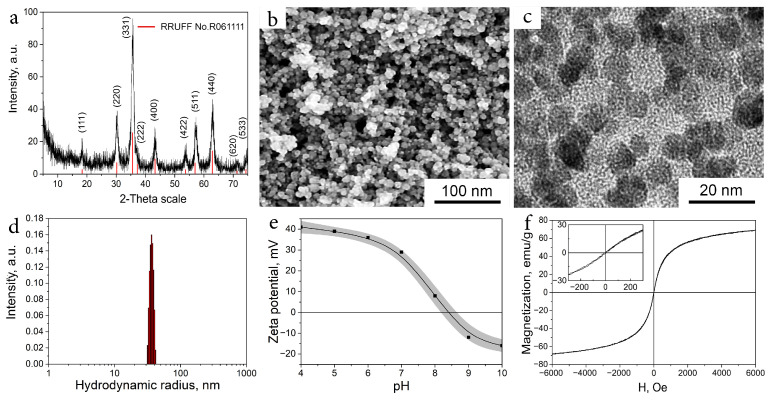
Characterization of magnetic cores. XRD pattern of Ferria (**a**); SEM image (**b**); TEM image (**c**); DLS characterization of Ferria (**d**); zeta potential as a function of pH (**e**); magnetization curve of the material (**f**).

Magnetite nanoparticles (MNPs) that were chosen as a magnetic component of the system were obtained by the coprecipitation of a non-stochiometric mixture of Fe(II) and Fe(III) chlorides with ammonium hydroxide, brought to pH 7 and ultrasonically threatened as was described in our earlier works [26,27,28]. This process leads to a highly stable hydrosol of MNPs at neutral pH with a pristine surface of nanoparticles, which is known as Ferria [26]. The mean crystallite size of the synthesized MNPs according to X-ray diffraction (XRD) analysis was 10.3 nm (Figure 1a). It was additionally proved by SEM and TEM imaging (Figure 1b–c). The XRD pattern of the material showed the main peak at 35.58${}^{\circ }$ corresponded to the crystalline plane with Miller indices of (311) as well as other distinctive peaks at 18.36${}^{\circ }$ (111), 30.10${}^{\circ }$ (220), 43.25${}^{\circ }$ (400), 53.66${}^{\circ }$ (422), 57.20${}^{\circ }$ (511), 62.82${}^{\circ }$ (440), 71.28${}^{\circ }$ (620), and 74.33${}^{\circ }$ (533) that are typical for magnetite crystalline phase (RRUFF No.R061111). According to dynamic light scattering (DLS) data, the mean hydrodynamic radius of the MNPs was 34 nm, and the zeta potential reached +29 mV (Figure 1d). As was investigated earlier, such a high value of zeta potential of the synthesized system originates from Fe(II)-OH groups on the surface of the MNPs that shift the isoelectric point of the material to pH 8.2 (Figure 1e) [26,29]. The magnetization curve of the particles demonstrated superparamagnetic behavior of the material and absence of remnant magnetization without external magnetic fields (Figure 1f).

### 2.2. Biomimetic Modification of MNPs

**Figure 2:**
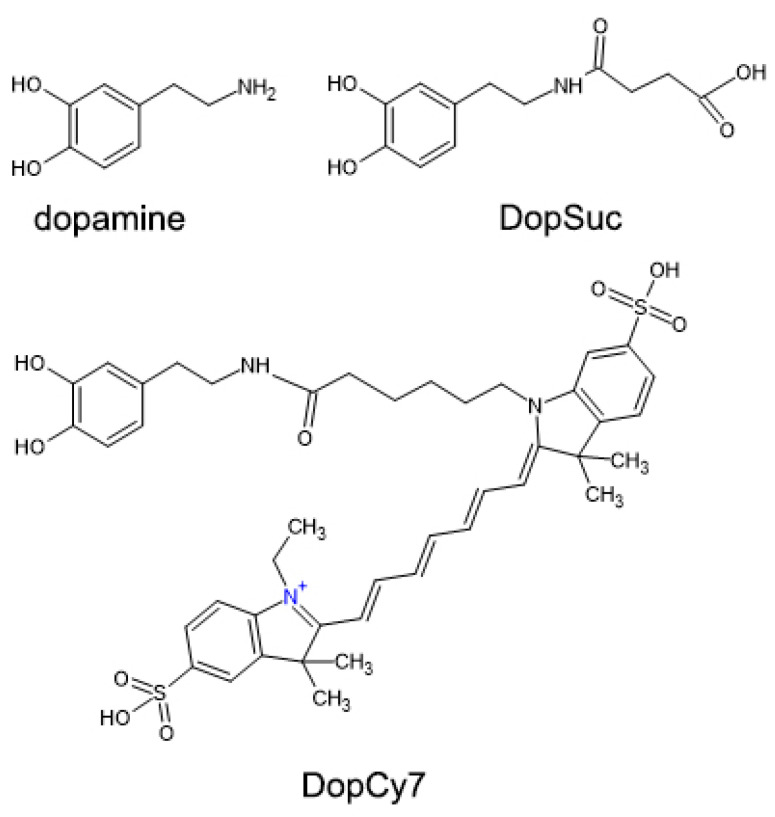
Structures of dopamine and dopamine derivatives used in this study.

**Figure 3:**
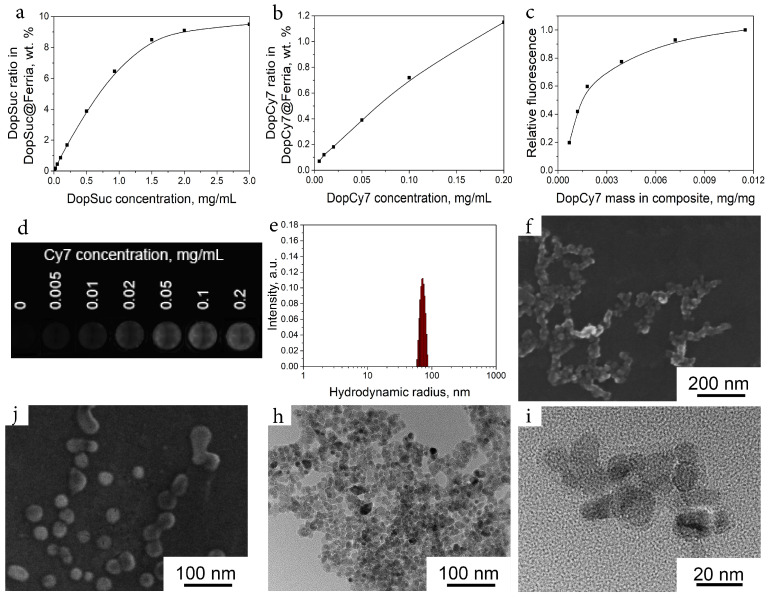
Modification of Ferria with DDs. Mass fraction of the coating in the composite as a function of DopSuc (**a**) or DopCy7 (**b**) concentration in solution; relative fluorescence of the composite as a function of DopCy7 mass fraction in its composition (**c**); visual appearance of the fluorescent composites (λ${}_{ex}$ = 730 nm, λ${}_{em}$ = 780 nm) depending on the used concentration of DopCy7 during deposition step (**d**); the hydrodynamic radius of the composite measured by DLS technique (**e**); SEM images of the composite (**f**,**j**); TEM images of the material (**i**,**h**).

Modification of the MNPs was performed via biomimetic polymerization of dopamine derivatives (DDs). Two dopamine derivatives were used in this study, namely, 4-[[2-(3,4-dihydroxyphenyl)ethyl]amino]-4-oxobutanoic acid (DopSuc) and N-Cyanine-7-3,4-dihydroxyphenethylamine (DopCy7) which were synthesized by acylation of dopamine via conventional organic synthetic protocols (see Figure 2 and Materials and Methods for details). A DopSuc derivative was selected as a cheap and synthetically available derivative suitable for general surface `’carboxylation”, while the more expensive DopCy7 derivative was chosen as a potential micro-component to introduce a fluorescent motif in the composite. For the biomimetic modification process, Ferria was mixed with a methanolic solution of organic molecules, and the pH of the system was brought to 8.5 with a phosphate buffer. The immobilization efficiency for both dopamine derivatives had a non-linear dependency on the MNPs:DD mass ratio as demonstrated in Figure 3a,b. The efficiency of Ferria’s surface modification with DopSuc was investigated in a wide range of concentrations up to 3 mg/mL. With the increase of DopSuc concentration in the reaction mixture, the mass fraction of the derivative in the formed DopSuc@Ferria reached its limit near 10% wt. at high concentrations of the derivative (Figure 3a). The efficiency of DopCy7 adsorption was linearly dependent on its concentration (Figure 3b), but at the same time, the intensity of the fluorescence signal showed non-linear dependence on the mass fraction of the DopCy7 in the DopCy7@Ferria composite, probably due to self-quenching effects (Figure 3c,d). For the creation of multifunctional hybrid composites, both DDs were immobilized simultaneously from the mixture of DopSuc:DopCy7, resulting in bifunctional material DopSucCy7@Ferria. After absorption of the organic layer, the hybrid material had a hydrodynamic radius of 72 nm and a zeta potential of -15 mV at pH 7 (Figure 3e). The hybrid material had a spherical shape according to SEM and TEM images, with a diameter of individual particles near 23 ± 5 nm (Figure 3f–i). Taking into consideration DLS data, it could be supposed that in a colloidal solution these particles are presented in a form of loosely associated aggregates that can be separated under ultrasonication to form a monolayer of individual hybrid nanoparticles.

**Figure 4:**
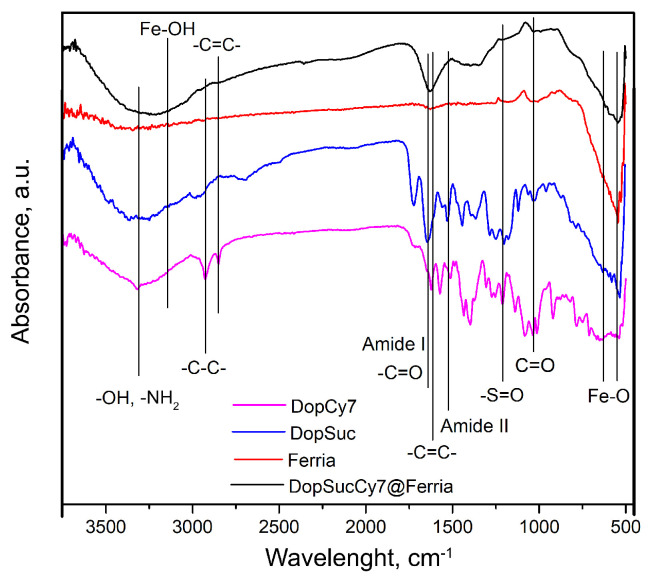
FTIR spectra of the synthesized composite.

Investigation of the DopSucCy7@Ferria by Fourier-transform infrared spectroscopy (FTIR) was carried out to characterize the organo–inorganic composites and confirm the modification of MNPs. The results are presented in Figure 4. While a detailed description of the material is impossible due to the intensity of the signals from the composite, it is still possible to determine the characteristic functional groups on its surface. Strong bands near 550 cm${}^{-1}$ with a shoulder at 630 cm${}^{-1}$ that correspond to Fe-O vibration and a broad band at 3000-3300 cm${}^{-1}$ due to hydroxyl groups from adsorbed water molecules and Fe-OH groups are noted [30]. Much more pronounced broadband at 3300–3500 cm${}^{-1}$ can be attributed to characteristic stretching vibrations of -OH and -NH${}_{2}$ groups on the surface of the material, while shoulders near 2940 and 2850 cm${}^{-1}$ are due to aliphatic and aromatic carbons in its structure. A broad peak near 1642 cm${}^{-1}$ is a combination of bands from the Amide I band and signals from the carboxylic -C=O group, overlaid by -C=C stretching vibrations near 1614 cm${}^{-1}$ and the Amide II band at 1525 cm${}^{-1}$. The abovementioned signals are accompanied by the peak at 1037 cm${}^{-1}$ corresponding to -C=O vibrations of the amide group [31]. A weak band near 1215 cm${}^{-1}$ corresponding to -S=O stretching in the –SO${}_{3}$H group of Cy7 residue can be observed in the spectra of the composite material [32].

### 2.3. Bioactivity of Nanocomposite

**Figure 5:**
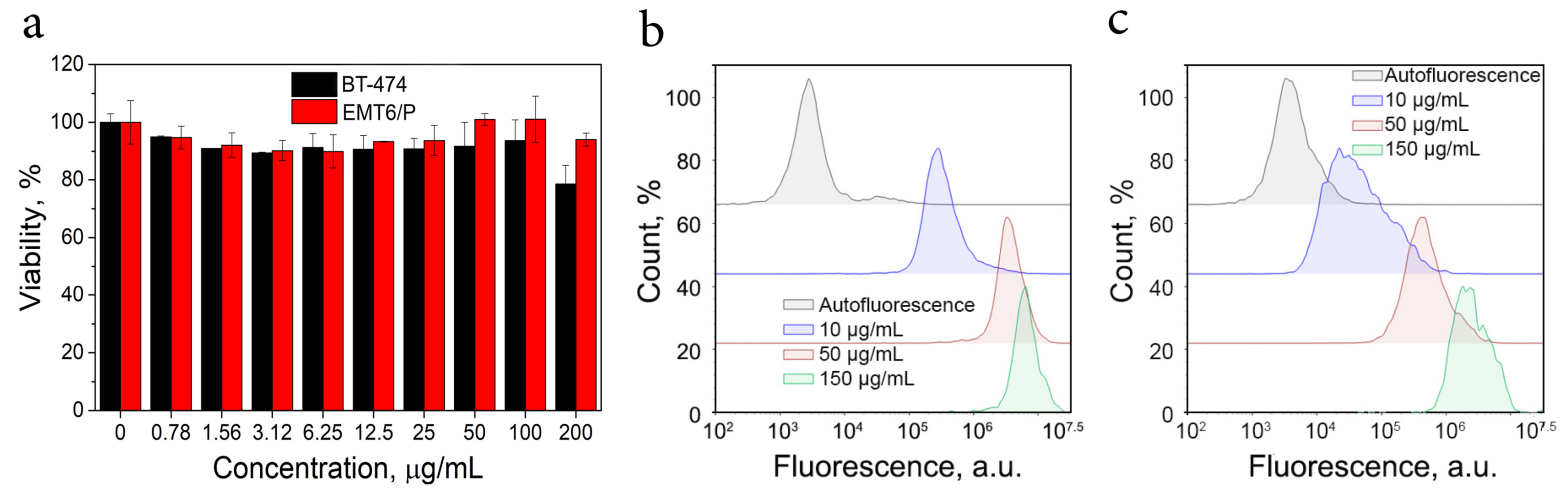
In vitro activity of Ab-DopSycCy7@Ferria composite. Cytotoxicity of the hybrid material measured on BT-474 and EMT6/P cell lines (**a**); flow cytometry fluorescence signal of BT-474 cells labeled with the composite (**b**); flow cytometry fluorescence signal of EMT6/P cells labeled with the composite at various concentrations (**c**).

Biocompatibility of the synthesized material was accessed for in vitro and in vivo applications. Due to the presence of carboxylic groups on the surface of the composite, it could be modified via conventional biochemical approaches, for example by carbodiimide chemistry. To evaluate this possibility, the particles were modified with trastuzumab, a well-known antibody against human epidermal growth factor receptor 2 (HER2) which is a specific cancer marker [33] (Ab-DopSucCy7@Ferria). The number of antibodies conjugated to composite was measured by BCA protein assay and was found at the level of 0.42% wt. Biocompatibility of the synthesized system was evaluated on a human ductal carcinoma of the breast cell line expressing HER2 receptor (BT-474) and on the mouse mammary cell line where this receptor was absent (EMT6/P). The hybrid material showed no cytotoxic effects in a wide range of concentrations up to 200 μg/mL after incubation for 72 h (Figure 5a).

The cell-targeting efficiency was evaluated on HER2-overexpressing cells BT-474 using flow cytometry. An as-synthesized Ab-DopSucCy7@Ferria composite was incubated with HER2+ and HER2- cells, and the selectivity of the hybrid material was measured by calculating the fluorescent signal. The flow cytometry results demonstrated that antibody-conjugated material specifically interacted with HER2+ BT-474 cells in a concentration-dependent manner and possessed a much lower affinity toward the EMT6/P cell line which lacks a HER2 receptor (Figure 5b,c). The composite had 8.2 and 8.0 times higher affinity toward HER2 positive cell lines than to HER2 negative ones at low concentrations valued at 10 and 50 μg/mL, respectively, but this difference reduced to 2.7 folds at 150 μg/mL.

**Figure 6:**
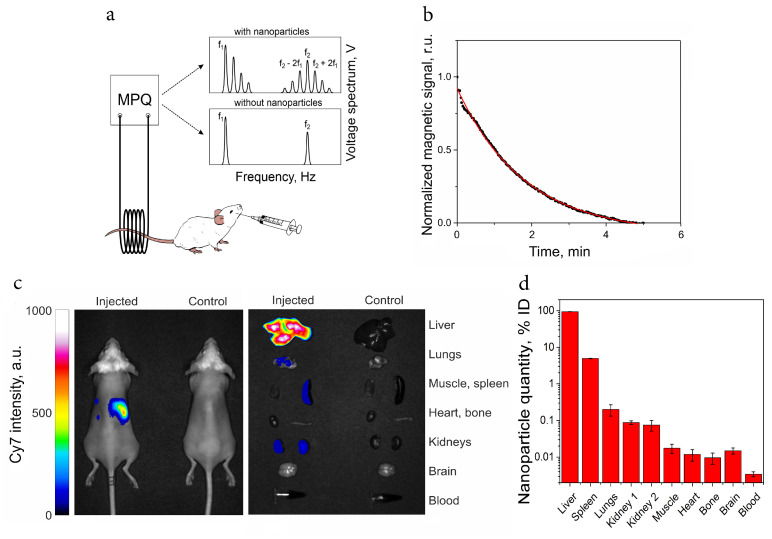
Circulation time and biodistribution of the composite in mice. Schematic representation of circulation dynamics measurements with MPQ device (**a**); representative circulation kinetics for 300 μg of DopSucCy7@Ferria measured by the MPQ method. Black dots correspond to the magnetic signal of the device at certain time points; the red curve corresponds to the exponential fitting curve (**b**); in vivo and ex vivo fluorescent images superposed with bright field images of a control animal (right) and injected with DopSucCy7@Ferria (left), λ${}_{ex}$ = 730 nm and λ${}_{em}$ = 780 nm (**c**); biodistribution of the composite measured with the MPQ technique (**d**).

In vivo biodistribution and circulation time of the nanoparticles were evaluated in BALB/c mice. For circulation time measurements, the magnetic composite was injected via retro-orbital sinus, and the magnetic signal was monitored in the tail vein with the magnetic particle quantification (MPQ) technique which is described in detail elsewhere [34]. Briefly, this method is based on the response of materials to a combination of two alternating magnetic fields with frequencies *f${}_{1}$* and *f${}_{2}$*. For such systems, the response is recorded at combinatorial frequencies *f = nf${}_{1}$± mf${}_{2}$*, where *n* and *m* are integers (Figure 6a). At these conditions, only non-linear magnetic materials such as superparamagnetic nanoparticles contribute to the signal, while signals from dia- and paramagnetic are absent, so there is no background noise and a good signal-to-noise ratio. MPQ features a linear dynamic range of seven orders of magnitude and offers time resolution in a seconds range [35]. The tested composite showed a circulation half-life of 1.40 ± 0.56 mim, n = 3 (Figure 6b).

For evaluation of the composite biodistribution profile, mice were injected with 300 μg of composite, and the fluorescent and magnetic signals were recorded by the optical imaging method and the MPQ method, respectively (Figure 6c,d). The results revealed that the composite was located mainly in the liver (94.58 ± 0.15% of injected dose) and spleen (5.00 ± 0.06%). A relatively small proportion of nanoparticles was also found in the lungs and kidneys.

## 3. Discussion

**Figure 7:**
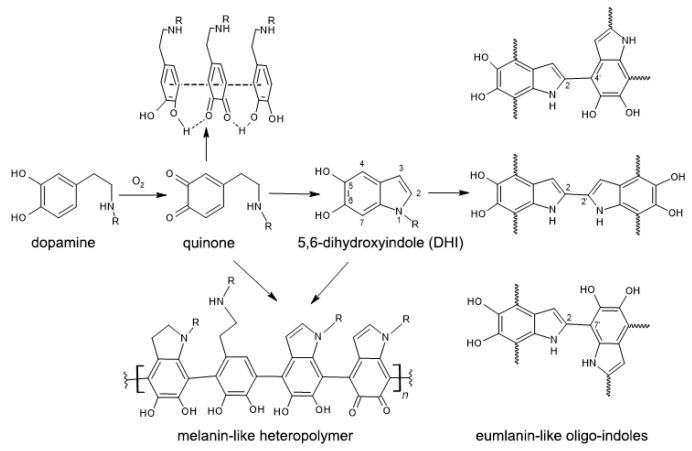
Schematic representation of dopamine polymerization, reproduced from [37].

Here, we described the potency of the biomimetic surface modification strategy with dopamine derivatives for the production of bio-applicable multifunctional systems. This strategy is based on oxidative polymerization of dopamine and dopamine derivatives under mild conditions that result in in situ formation of organic coatings on a substrate. While the exact mechanism of the occurring process remains unknown and under intense investigation, this approach can be widely used for the creation of various nanostructured materials and systems. It is accepted that the mechanism of dopamine polymerization involves its oxidation by dissolved oxygen into quinone intermediates that undergo intramolecular cyclization resulting in 5,6-dihydroxyindole [17,36]. These three compounds could interact by covalent inter-molecular bonding and form eumelanin- and melanin-like structures as well as by non-covalent self-assembly through hydrogen bonds π–π stacking or hydrophobic interactions (Figure 7. The formed complex mixture of products can deposit on a variety of substrates to form organic coating even on inert surfaces. In the case of magnetite nanoparticles, slightly acidic phenolic hydroxyls of dopamine and its derivatives interact with under-coordinated iron on the surface of the nanoparticle back to its octahedral bulk-like lattice structure.

In our study, we selected two compounds, DopSuc and DopCy7, with different roles. The first one was selected as a synthetically available compound to introduce usable carboxyl groups to the surface of inorganic material. DopCy7 derivative was taken as a model compound due to its spectral characteristic. Its excitation and emission peaks lie in the near-infrared region in the so-called near-infrared window of tissue transparency. This molecule was designed for in vivo imaging in biological experiments, and its conjugation to magnetic nanoparticles potentially led to the production of bifunctional magneto-optical diagnostic agents by biomimetic modification process.

It was shown earlier that the effectiveness of the whole process of PDA formation is dependent on the rates of eumelanin- and melanin-like polymers formation, their nucleation, and deposition of the substrate’s surface [16,19]. A higher pH level elevates the rate of oxidized dopamine product formation, but at the same time, high pH leads to deprotonation of phenolic hydroxyls which have pK${}_{a}$ 9.0–9.5. Ionization of these compounds improves the solubility of the formed derivatives and favors the dissolution of the polymer rather than its adsorption to the surface [12]. In addition, the negative charge of the surface inhibits the rate of adsorption of the dissociated catechol groups which leads to lower nucleation and deposition rates. In our case, this effect resulted in the limitation of DopSuc adsorption efficiency on the level of 0.095 mg per mg of Ferria particles due to the enrichment of the surface with carboxylic groups. At the same time, this issue had a negligible effect on the process of DopCy7 deposition as this compound was not meant to be adsorbed at high concentrations and was used as a micro-component of the hybrid material.

Biomimetic polymerization of DopSuc and DopCy7 from their mixture led to the formation of a hybrid bifunctional layer with carboxylic groups and fluorescent motifs at the same time. This strategy could potentially be used for the introduction of more than two functional groups simultaneously and will be investigated in detail in our further works.

The initial purpose of the study was to evaluate the potency of the biomimetic surface modification strategy for the creation of bio-applicable systems. One of the main criteria for the bioapplicability of materials is their bioactivity and toxicity. The synthesized composite material was based on an iron oxide core, namely magnetite. Magnetite is known for its good biocompatibility and can be easily metabolized in the body. Moreover, it is the only magnetic material approved for parenteral administration [38]. The MNPs used in this study were synthesized by the co-precipitation method without any surfactants or surface modification agents with possible toxicity profiles [39]. Biomimetic coatings based on dopamine derivatives are synthesized under mild conditions without any aggressive or toxic compounds and dopamine in its turn is a biogenic molecule. The combination of these factors ensured low cytotoxicity of the material which was proved by in vitro studies even at high concentrations of the agent. At the same time, the presence of carboxylic groups on its surface allowed conjugation with recognizing bioligands such as antibodies against HER2 to render the nanocomposite selective to cancer cells. The amount of the conjugated antibody was assessed by the BCA protein assay and showed that the final composite had 0.42% wt. of antibodies in its composition. This corresponds to approximately 5–25 antibodies per nanoparticle or about one antibody per 2000 nm${}^{2}$ of the surface. The obtained grafting density was typical for an unoptimized carbodiimide coupling strategy and suitable for further initial experiments, while it should be mentioned that other conjugation approaches may give much higher modification degrees [40,41].

Selectivity of the antibody-conjugated nanoparticles toward a corresponding antigen was evaluated on HER2-expressing and nonexpressing cell lines BT-474 and EMT6/P respectively. It was found that at concentrations up to 50 μg/mL the material was highly selective toward the targeted receptor, but at higher concentrations, its selectivity was lowered. To explain this result, it is necessary to take into account that the highly specific interaction of trastuzumab-equipped nanoparticles with HER2-overexpressing cells is accompanied by different types of non-covalent non-specific interactions, namely, ionic interactions, hydrophobic interactions, and hydrogen bonding, since the anti-HER2 nanoparticles are added to HER2-overexpressing cells in a significant excess relative to the number of HER2 receptor molecules on the cell surface. With the increased concentrations of nanoparticles, all nanoparticle binding sites due to HER2-trastuzumab interactions became occupied. From this moment, non-specific interactions prevail over specific ones, which leads to decreased binding selectivity. Such behavior is typical for nanoformulations and correlates with previously published data [42].

In vivo behavior of nanomaterials and nanosystems was mainly determined by their circulation time in the bloodstream and biodistribution profile after retro-orbital injection. Injection into the retroorbital sinus (the ophthalmic plexus route) is actively used in various areas of in vivo research for intravenous injection in mice along with injection into the lateral tail vein [43,44]. In our work, this method was preferable, since it avoided damage to the tail, which was then placed in the coil of the MPQ device. In addition, the outlying place of injection prevented instantaneous deposition of magnetic particles in tail veins that could interfere with circulation kinetics detection. The tested composite showed a circulation half-life of approximately 1.5 min which is typical for nanoparticles without anti-biofouling coatings. It is known from the literature that upon systemic injection into the bloodstream, nanoparticles are surrounded by blood plasma proteins, some of which are opsonins, while some are dysopsonins. These proteins form rigid and soft coronas determining nanoparticle behavior in the organism and triggering their rapid opsonization by macrophages [45,46]. To increase a circulation time, additional modifications of the composite’s surface by hydrophilic coatings, such as polyethylene glycol derivatives, polysaccharides, or zwitterionic polymers [47,48], can be used. Alternatively, the process of opsonization can be inhibited by induced eryptosis or macrophage blockade with ballast nanoparticles [49,50]. These powerful techniques showed their efficiency in prolongation of blood circulation time on a variety of nanosized systems and may be applied without additional modification of the surface of the nanoparticles.

The biodistribution profile from both optical visualization and MPQ studies showed that particles are mainly located in the liver and spleen—organs that play a major role in nanoparticle clearance. It was described earlier that Kupffer cells in the liver and splenic macrophages react rapidly to the protein corona that is formed on injected nanoparticles unless the surface of the letter is protected from protein adsorption. Thus, the observed biodistribution correlates with the literature data [33,50].

## 4. Materials and Methods

*Chemicals:* All reagents and solvents were obtained or distilled according to standard procedures. Sodium phosphate dibasic dihydrate, Tris base, sulfo-N-hydroxysuccinimide (sulfo-NHS), N-(3-Dimethylaminopropyl)-N’-ethylcarbodiimide hydrochloride (EDC), sodium phosphate monobasic monohydrate, dimethyl sulfoxide (DMSO), pyridine, dopamine hydrochloride, triethylamine and succinic anhydride were obtained from Sigma Aldrich (St. Louis, MO, USA). Cyanine7-NHS activated ester was obtained from Lumiprobe (Moscow, Russia). HER2 antibodies (Trastuzumab) were obtained from Genentech (South San Francisco, CA, USA).

*Cell culture:* Mouse mammary cell line EMT6/P and a human mammary cell line with a stable expression of HER2 receptor BT-474 were cultured in DMEM medium (HyClone, Logan, UT, USA) supplemented with 10% fetal bovine serum (HyClone, Logan, UT, USA), penicillin/streptomycin (PanEko, Moscow, Russia), and 2 mM L-glutamine (PanEko, Moscow, Russia). Cells were incubated under a humidified atmosphere with 5% CO${}_{2}$ at 37 ${}^{\circ }$C.

*Synthesis of magnetite nanoparticles (**Ferria**):* Stable magnetite hydrosol was prepared by the procedure described earlier [51,52]. Briefly, 2.5 g FeCl${}_{2}$*4H${}_{2}$O and 5 g FeCl${}_{3}$*6H${}_{2}$O were dissolved in 100 mL of deionized water. Then, 11 mL of 30% NH${}_{4}$OH was added under constant stirring (500 rpm) at room temperature. The formed magnetite precipitate was magnetically separated and washed with deionized water until neutral pH. The washed black precipitate was mixed with 100 mL of deionized water and subjected to ultrasonic treatment (37 kHz, 110 W) under constant stirring (300 rpm). The resulting sol was diluted with deionized water to a mass fraction of MNPs of 1% wt. The stock hydrosol was stored at 4 ${}^{\circ }$C.

For *4-[[2-(3,4-dihydroxyphenyl)ethyl]amino]-4-oxobutanoic acid (DopSuc):* To a solution of 1.89 g, (10 mmol) dopamine hydrochloride in 20 mL of pyridine 1.05 g (10.5 mmol) of succinic anhydride was added under nitrogen atmosphere. The solution was stirred at room temperature overnight. After that, the mixture was evaporated to 5 mL, mixed with 0.1 M HCl, and extracted with ethylacetate (4 × 20 mL). The organic phase was washed with 0.1 M HCl, water, and brine, dried over sodium sulfate, and evaporated. The residue was crystallized from isopropanol to give a white solid. (1.51 g, 60% yield), mp 128–129 ${}^{\circ }$C.

${}^{1}$H NMR (300 MHz, DMSO-d6) σ 8.2 (1H, s, NH), 7.58 (1H, s, ArOH), 6.87 (1H, s, ArOH), 6.70 (1H, d, J = 9 Hz, ArH), 6.54 (1H, m ArH), 6.47 (1H, m, ArH), 3.25 (2H, m, CH2-NH), 2.68 (2H, t, J = 8.7 Hz, CH2-COOH), 2.58–2.48 (4H, m); ${}^{13}$C NMR (125 MHz, DMSO-d6) σ 174.61, 173.12, 143.88, 143.56, 131.12, 119.64, 116.76, 115.50, 40.16, 36.29, 30.10, 27.12. HRMS for C${}_{12}$H${}_{15}$NO${}_{5}$ [M+H]${}^{+}$ Calc.: 252.0877, found: 252.0870.

*N-Cyanine-7-3,4-dihydroxyphenethylamine (DopCy7):* 19 mg (0.1 mmol) of dopamine hydrochloride and 14 μL (10 mg, 0.1 mmol) of triethylamine was dissolved in 1 mL of degassed DMF under argon atmosphere. After that, 73 mg of Cy7-NHS ester was added to the solution, and the reaction mixture was stirred for 12 hours in darkness. The resulting solution was poured into water and extracted with chloroform (3 × 5 mL). Organic extracts were washed with 0.1 M HCl, water, and brine, and evaporated in vacuo. The obtained material was recrystallized from methanol to give 32 mg of black-purplish product with 40% yield, mp 140–1142 ${}^{\circ }$C.

${}^{1}$H NMR (300 MHz, DMSO-d6) σ 8.00 (1H, m), 7.92 (1H, m), 7.87–7.80 (3H, m), 7.71–7.62 (2H, m), 7.57 (1H, m), 7.49 (1H, m), 7.26 (1H, m), 7.04 (1H, d, J = 7.9 Hz), 6.85–6.44 (7H, m), 6.34 (1H, m), 6.11 (1H, m), 4.19 (2H, s), 3.83 (2H, m), 3.17 (2H, m), 2.15 (2H, m), 1.74 (9H, m), 1.61–1.39 (10H, m). ${}^{13}$C NMR (125 MHz, DMSO-d6) σ 173.09, 167.57, 158.97, 150.01, 148.99, 146.75, 144.55, 143.21, 142.81, 141.37, 138.14, 137.31, 136.70, 134.58, 133.01, 132.04, 130.68, 129.05, 128.86, 124.10, 122.63, 122.04, 115.80, 115.21, 114.83, 114.12, 112.34, 100.33, 45.81, 45.60, 41.53, 41.25, 37.47, 34.50, 34.49, 28.09, 27.25, 27.16, 26.48, 24.43. HRMS for C${}_{42}$H${}_{50}$N${}_{3}$O${}_{9}$S${}_{2}$ [M+H]${}^{+}$ Calc.: 805.3061, found: 805.0142

*Biomimetic modification of MNPs:* For PDA layer formation experiments, 100 μL of Ferria hydrosol was mixed with 100 μL of dopamine derivative solution of the desired concentration in methanol and ultrasonically treated for 10 min. After that, 200 μL of 0.05 M phosphate buffer with pH 8.5 was added, and the system was ultrasonicated for another 15 min. The mixture was incubated for 24 h on a rotator. Hybrid material was magnetically separated, washed six times with 1 mL of deionized water, diluted with 100 μL of water, and stored at 4 ${}^{\circ }$C. The collected liquid fractions were combined and analyzed with UV spectroscopy at 260 nm. The amount of adsorbed DDs derivatives was calculated as a difference between the obtained and theoretical values.

*Conjugation with HER2 antibodies:* HER2 antibodies were conjugated with the composite by carbodiimide strategy; 1 mg of the material in 100 μL of water was mixed with 12 mg of EDC and 2 mg of sulfo-NHS dissolved in 60 μL of 0.1 M MES (2-(N-morpholino) ethanesulfonic acid), pH 5.0 buffer. The resulting mixture was ultrasonicated and incubated for 30 min. After that, magnetic material was separated with a magnet and mixed with 80 μL of HER2 antibodies solution in PBS with 1 g/L concentration to produce Ab-DopSucCy7. The mixture was incubated for 8 h at room temperature and then triple washed with 200 μL of PBS-1% bovine serum albumin (BSA) buffer and resuspended in 100 μL PBS-1% BSA.

*Efficiency of antibodies conjugation:* The number of antibodies conjugated to the hybrid material was measured by the BCA protein assay kit (Abisense LLC, Sochi, Russia). A total of 50 μL of Ab-DopSycCy7@Ferria composite solution with a concentration of 10 g/L was mixed with 150 μL of BCA working reagent and was incubated for 20 min. The composite was magnetically separated, and the optical density of the supernatant was measured at 540 nm using a plate reader. DopSycCy7@Ferria was treated in the same manner and used in a control experiment. The protein concentration was calculated using the BSA calibration curve. The approximate number of conjugated antibodies per nanoparticle was calculated by dividing the number of protein molecules $N_{Ab}$ by the approximate number of nanoparticles $N_{comp}$. The latter was calculated by the Equation (1):

(1) $$N_{comp}=\frac{m_{composite}}{4/3\pi R^{3}*\rho_{Fe_{3}O_{4}}}$$

where $m_{composite}$ is a mass of composite, *R* is the radius of nanoparticles (20 or 72 nm), and $\rho_{Fe_{3}O_{4}}$ is a density of magnetite (4.82 g/${\mathrm{cm}}^{3}$).

The approximate density of antibody conjugation (D) was calculated by Equation (2):

(2) $$D=\frac{N_{Ab}}{4\pi R^{2}*N_{comp}}$$

*Cytotoxicity:* The cytotoxicity of NPs was investigated using the MTT test. EMT6/P and BT-474 cells were plated onto a 96-well plate at 2.5 × 10${}^{3}$ cells per well in 100 μL of DMEM medium with 10% FBS. After overnight culturing, 100 μL of either DMEM 10% FBS or DMEM 10% FBS with nanoparticles at various concentrations was added to the cells. The cells were incubated for 48 h in a 5% CO_2_ atmosphere at 37 ${}^{\circ }$C. Then, the medium was removed, and the cells were washed once with the medium. Next, 100 μL of 0.5 g/L MTT solution in serum-free DMEM medium was added to the wells and incubated for 1 h. Next, the MTT solution was removed, and 100 μL of DMSO was added to the wells. The optical density of wells was measured at a wavelength of λ = 570 nm.

*Flow cytometry:* For flow cytometry, Cyanine5-labeled nanoparticle conjugates were prepared by mixing 100 μg of Ab-DopSucCy7@Ferria nanoparticles (10 g/L in 100 μL of PBS) with 5 μg of Cy5-NHS ester in 5 μL of DMSO. The mixture was incubated for 10 min at room temperature and washed from unbound molecules thrice with PBS using a magnetic separator resuspended in 100 μL of PBS with 1% BSA. The resulting nanoparticles Cy5-Ab-DopSucCy7@Ferria were suitable for flow cytometer analysis by Novocyte 2000 VYB flow cytometer (ACEA Biosciences, San Diego, CA, USA).

To determine Cy5-Ab-DopSucCy7@Ferria cell-binding efficiency, the harvested EMT6/P and BT-474 cells were washed with PBS, resuspended in 300 μL of PBS with 1% BSA at a concentration of 1 × 10${}^{6}$ cells/mL, incubated with Cy5-Ab-DopSucCy7@Ferria at different concentrations for 15 min, then washed from unbound particles by centrifugation for 3 min at 100×*g*, resuspended in 100 μL PBS, and analyzed using a Novocyte 2000R flow cytometer in a fluorescence channel suitable for Cy5 detection (excitation laser 640 nm, emission filter 675/30 nm).

*Circulation half-life studies:* Female BALB/c mice of 24–26 g weight were used for in vivo studies. Some 300 μg of magnetic particle suspension in 100 μL of PBS was administered retro-orbitally, and the dynamics in the tail were recorded with the MPQ technique. Half-life was calculated to characterize the behavior of the major portion of the injected dose by monoexponential fitting of the data points in the mid 80% signal range of the clearance curve. Experiments were conducted three times.

*Biodistribution studies:* In vivo and ex vivo fluorescent analysis of biodistribution was performed on a LumoTrace FLUO bioimaging system (Abisense LLC, Sochi, Russia) using λ${}_{ex}$ = 730 nm and λ${}_{em}$ = 780 nm with exposure time 300 ms. Experiments were conducted three times.

For the MPQ analysis of magnetic-particle biodistribution, the organs and tissues were extracted 1 h after nanoparticle injection and placed in the measuring coil of the MPQ detector. The integral magnetic signal from each organ was normalized to the total signal from all measured organs. Such data were then averaged between three different animals.

## 5. Conclusions

Here, we described the application of the biomimetic dopamine polymerization approach for the creation of a multifunctional magnetic fluorescent composite in a single step from a mixture of dopamine derivatives. Magnetic nanoparticles were modified with dopamine conjugates under mild conditions, leading to multifunctional organo–inorganic composites. The synthesized material showed excellent biocompatibility and a typical biodistribution profile. The contingency of the modification process and bioactivity of the synthesized materials proved the potency of the proposed strategy, which will be further evaluated in upcoming works.

## References

[1] Ventola C.L. (2017). Progress in nanomedicine: Approved and investigational nanodrugs. Pharm. Ther..

[2] Choi Y.H., Han H.K. (2018). Nanomedicines: CURRENT status and future perspectives in aspect of drug delivery and pharmacokinetics. J. Pharm. Investig..

[3] Huang L., Zhao S., Fang F., Xu T., Lan M., Zhang J. (2021). Advances and perspectives in carrier-free nanodrugs for cancer chemo-monotherapy and combination therapy. Biomaterials.

[4] Drozdov A.S., Nikitin P.I., Rozenberg J.M. (2021). Systematic review of cancer targeting by nanoparticles revealed a global association between accumulation in tumors and spleen. Int. J. Mol. Sci..

[5] Singh D., Dilnawaz F., Sahoo S.K. (2020). Challenges of moving theranostic nanomedicine into the clinic. Nanomedicine.

[6] Martins J.P., Das Neves J., de la Fuente M., Celia C., Florindo H., Günday-Türeli N., Popat A., Santos J.L., Sousa F., Schmid R. (2020). The solid progress of nanomedicine. Drug Deliv. Transl. Res..

[7] Gadekar V., Borade Y., Kannaujia S., Rajpoot K., Anup N., Tambe V., Kalia K., Tekade R.K. (2021). Nanomedicines accessible in the market for clinical interventions. J. Control. Release.

[8] Mu Q., Yu J., McConnachie L.A., Kraft J.C., Gao Y., Gulati G.K., Ho R.J. (2018). Translation of combination nanodrugs into nanomedicines: Lessons learned and future outlook. J. Drug Target..

[9] Wang X., Hou Y., Yao L., Gao M., Ge M. (2016). Generation, characterization, and application of hierarchically structured self-assembly induced by the combined effect of self-emulsification and phase separation. J. Am. Chem. Soc..

[10] Vinogradov V.V., Drozdov A.S., Mingabudinova L.R., Shabanova E.M., Kolchina N.O., Anastasova E.I., Markova A.A., Shtil A.A., Milichko V.A., Starova G.L. (2018). Composites based on heparin and MIL-101 (Fe): The drug releasing depot for anticoagulant therapy and advanced medical nanofabrication. J. Mater. Chem. B.

[11] Zhong X., Yang K., Dong Z., Yi X., Wang Y., Ge C., Zhao Y., Liu Z. (2015). Polydopamine as a biocompatible multifunctional nanocarrier for combined radioisotope therapy and chemotherapy of cancer. Adv. Funct. Mater..

[12] Chen Z., Wang Z., Gu Z. (2019). Bioinspired and biomimetic nanomedicines. Acc. Chem. Res..

[13] De Oliveira H., Thevenot J., Lecommandoux S. (2012). Smart polymersomes for therapy and diagnosis: Fast progress toward multifunctional biomimetic nanomedicines. Wiley Interdiscip. Rev. Nanomed. Nanobiotech..

[14] Drozdov A., Prilepskii A., Koltsova E., Anastasova E., Vinogradov V. (2020). Magnetic polyelectrolyte-based composites with dual anticoagulant and thrombolytic properties: Towards optimal composition. J. Sol-Gel Sci. Technol..

[15] Lee H., Dellatore S.M., Miller W.M., Messersmith P.B. (2007). Mussel-inspired surface chemistry for multifunctional coatings. Science.

[16] Qiu W.Z., Yang H.C., Xu Z.K. (2018). Dopamine-assisted co-deposition: An emerging and promising strategy for surface modification. Adv. Colloid Interface Sci..

[17] Ryu J.H., Messersmith P.B., Lee H. (2018). Polydopamine surface chemistry: A decade of discovery. ACS Appl. Mater. Interfaces.

[18] Klosterman L., Riley J.K., Bettinger C.J. (2015). Control of heterogeneous nucleation and growth kinetics of dopamine-melanin by altering substrate chemistry. Langmuir.

[19] Lee H.A., Park E., Lee H. (2020). Polydopamine and its derivative surface chemistry in material science: A focused review for studies at KAIST. Adv. Mater..

[20] Liebscher J., Mroówczynński R., Scheidt H.A., Filip C., Haădade N.D., Turcu R., Bende A., Beck S. (2013). Structure of polydopamine: A never-ending story?. Langmuir.

[21] Ball V. (2018). Polydopamine nanomaterials: Recent advances in synthesis methods and applications. Front. Bioeng. Biotechnol..

[22] Palladino P., Bettazzi F., Scarano S. (2019). Polydopamine: Surface coating, molecular imprinting, and electrochemistry—Successful Applications and future perspectives in (bio) analysis. Anal. Bioanal. Chem..

[23] Mazur M., Barras A., Kuncser V., Galatanu A., Zaitzev V., Turcheniuk K.V., Woisel P., Lyskawa J., Laure W., Siriwardena A. (2013). Iron oxide magnetic nanoparticles with versatile surface functions based on dopamine anchors. Nanoscale.

[24] Barclay T.G., Hegab H.M., Clarke S.R., Ginic-Markovic M. (2017). Versatile surface modification using polydopamine and related polycatecholamines: Chemistry, structure, and applications. Adv. Mater. Interfaces.

[25] Lee H.A., Ma Y., Zhou F., Hong S., Lee H. (2019). Material-independent surface chemistry beyond polydopamine coating. Accounts Chem. Res..

[26] Drozdov A.S., Ivanovski V., Avnir D., Vinogradov V.V. (2016). A universal magnetic ferrofluid: Nanomagnetite stable hydrosol with no added dispersants and at neutral pH. J. Colloid Interface Sci..

[27] Anastasova E.I., Puzyrev D., Ivanovski V., Drozdov A.S. (2020). Magnetically assisted synthesis of porous sol-gel magnetite matrices with structural anisotropy. J. Magn. Magn. Mater..

[28] Zakharzhevskii M., Drozdov A.S., Kolchanov D.S., Shkodenko L., Vinogradov V.V. (2020). Test-system for bacteria sensing based on peroxidase-like activity of inkjet-printed magnetite nanoparticles. Nanomaterials.

[29] Anastasova E.I., Ivanovski V., Fakhardo A.F., Lepeshkin A.I., Omar S., Drozdov A.S., Vinogradov V.V. (2017). A pure magnetite hydrogel: Synthesis, properties and possible applications. Soft Matter.

[30] Ivanovski V., Shapovalova O.E., Drozdov A.S. (2022). Structural Rearrangements of Carbonic Anhydrase Entrapped in Sol-Gel Magnetite Determined by ATR–FTIR Spectroscopy. Int. J. Mol. Sci..

[31] Kuznowicz M., Jędrzak A., Rębiś T., Jesionowski T. (2021). Biomimetic magnetite/polydopamine/*β*-cyclodextrins nanocomposite for long-term glucose measurements. Biochem. Eng. J..

[32] Yoshino F., Amano T., Zou Y., Xu J., Kimura F., Furusho Y., Chano T., Murakami T., Zhao L., Komatsu N. (2019). Preferential tumor accumulation of polyglycerol functionalized nanodiamond conjugated with cyanine dye leading to near-infrared fluorescence in vivo tumor imaging. Small.

[33] Lunin A.V., Korenkov E.S., Mochalova E.N., Nikitin M.P. (2021). Green Synthesis of Size-Controlled in Vivo Biocompatible Immunoglobulin-Based Nanoparticles by a Swift Thermal Formation. ACS Sustain. Chem. Eng..

[34] Zelepukin I.V., Yaremenko A.V., Ivanov I.N., Yuryev M.V., Cherkasov V.R., Deyev S.M., Nikitin P.I., Nikitin M.P. (2021). Long-term fate of magnetic particles in mice: A comprehensive study. ACS Nano.

[35] Nikitin M.P., Orlov A., Sokolov I., Minakov A., Nikitin P., Ding J., Bader S., Rozhkova E., Novosad V. (2018). Ultrasensitive detection enabled by nonlinear magnetization of nanomagnetic labels. Nanoscale.

[36] Cheng W., Zeng X., Chen H., Li Z., Zeng W., Mei L., Zhao Y. (2019). Versatile polydopamine platforms: Synthesis and promising applications for surface modification and advanced nanomedicine. ACS Nano.

[37] Volov A., Shkodenko L., Koshel E., Drozdov A.S. (2022). Bio-Inspired Surface Modification of Magnetite Nanoparticles with Dopamine Conjugates. Nanomaterials.

[38] Stueber D.D., Villanova J., Aponte I., Xiao Z., Colvin V.L. (2021). Magnetic nanoparticles in biology and medicine: Past, present, and future trends. Pharmaceutics.

[39] Andreeva Y., Drozdov A., Solovyeva A., Fakhardo A., Vinogradov V. (2020). Polyelectrolyte-based magnetic photonic crystals with anticoagulant activity. Mater. Today Chem..

[40] Montenegro J.M., Grazu V., Sukhanova A., Agarwal S., Jesus M., Nabiev I., Greiner A., Parak W.J. (2013). Controlled antibody/(bio-) conjugation of inorganic nanoparticles for targeted delivery. Adv. Drug Deliv. Rev..

[41] Marques A., Costa P., Velho S., Amaral M. (2020). Functionalizing nanoparticles with cancer-targeting antibodies: A comparison of strategies. J. Control. Release.

[42] Shipunova V., Nikitin M., Nikitin P., Deyev S. (2016). MPQ-cytometry: A magnetism-based method for quantification of nanoparticle–cell interactions. Nanoscale.

[43] Shimizu S. (2004). Routes of Adminstration. The Laboratory Mouse.

[44] Pinkerton W., Webber M. (1964). A method of injecting small laboratory animals by the ophthalmic plexus route. Proc. Soc. Exp. Biol. Med..

[45] Lundqvist M., Stigler J., Elia G., Lynch I., Cedervall T., Dawson K.A. (2008). Nanoparticle size and surface properties determine the protein corona with possible implications for biological impacts. Proc. Natl. Acad. Sci. USA.

[46] Zanganeh S., Spitler R., Erfanzadeh M., Alkilany A.M., Mahmoudi M. (2016). Protein corona: Opportunities and challenges. Int. J. Biochem. Cell Biol..

[47] Kim H., Röth D., Isoe Y., Hayashi K., Mochizuki C., Kalkum M., Nakamura M. (2021). Protein corona components of polyethylene glycol-conjugated organosilica nanoparticles modulates macrophage uptake. Colloids Surfaces Biointerfaces.

[48] Schöttler S., Landfester K., Mailänder V. (2016). Controlling the stealth effect of nanocarriers through understanding the protein corona. Angew. Chem. Int. Ed..

[49] Mirkasymov A.B., Zelepukin I.V., Nikitin P.I., Nikitin M.P., Deyev S.M. (2021). In vivo blockade of mononuclear phagocyte system with solid nanoparticles: Efficiency and affecting factors. J. Control. Release.

[50] Nikitin M.P., Zelepukin I.V., Shipunova V.O., Sokolov I.L., Deyev S.M., Nikitin P.I. (2020). Enhancement of the blood-circulation time and performance of nanomedicines via the forced clearance of erythrocytes. Nat. Biomed. Eng..

[51] Astafyeva B.V., Shapovalova O.E., Drozdov A.S., Vinogradov V.V. (2018). *α*-Amylase@ Ferria: Magnetic nanocomposites with enhanced thermal stability for starch hydrolysis. J. Agric. Food Chem..

[52] Rumyantceva V., Rumyantceva V., Andreeva Y., Tsvetikova S., Radaev A., Vishnevskaya M., Vinogradov V., Drozdov A.S., Koshel E. (2021). Magnetically controlled carbonate nanocomposite with ciprofloxacin for biofilm eradication. Int. J. Mol. Sci..

